# Hip dysplasia screening - epidemiological data from Valcea County

**Published:** 2016

**Authors:** RC Colta, C Stoicanescu, M Nicolae, S Oros, G Burnei

**Affiliations:** * Trauma and Orthopaedics Department, Central Military University Emergency Hospital, Bucharest; “Carol Davila” University of Medicine and Pharmacy, Bucharest, Romania; ** Paediatric Surgery Departament, Valcea County Emergency Hospital; Neonatal Department, Valcea County Emergency Hospital, Bucharest, Romania; *** C.I. Parhon National Institute of Endocrinology, Bucharest, Romania; **** Paediatric Trauma and Orthopaedic Department, “Marie Curie” Children Clinical Emergency Hospital, Bucharest, Romania

**Keywords:** congenital hip dislocation, perinatal risk factors, incidence, screening

## Abstract

Abstract

**Rationale:**:In its most severe form, developmental dysplasia of the hip (DDH) is one of the most common congenital malformations. Uncorrected developmental dysplasia of the hip is associated with long-term morbidity such as gait abnormalities, chronic pain, and degenerative arthritis.

**Aims:** (1) to identify the risks and predisposing factors involved in DDH (2) to define the incidence of the disease locally; (3) to emphasize the importance of ultrasonography in early diagnosis of DDH.

**Methods and results:**1021 newborns in the Neonatal Department of Valcea County Emergency Hospital were analyzed. The information contained in the observation sheets was collected and upon this, the ultrasound was performed accordingly to Graf’s classification. The following parameters were analyzed: sex, area of origin, gestational age, birthweight, type of delivery, parturition. Out of 1021 newborns, 27 had an abnormal ultrasound examination. 20 infants were diagnosed with DDH. Regarding the risk factors: cephalic delivery was associated with a lower risk/ the lowest of DDH (with p=0.045 for the right hip and p=0.001 for the left hip). Increased gestational age and increased birthweight were incriminating factors in the occurrence of DDH.

**Discussion:**Ultrasound imaging has become an accepted tool for accurately diagnosing DDH.

## Introduction

The definition of DDH is variable, including dislocated, dislocatable, subluxable and dysplastic hips. The development dysplasia of the hip is a leading cause of early arthritis and the most common congenital defect in the newborn with an estimated incidence ranging from 1 to 2 (1.2) per 1.000 live births [**1**]. The true incidence of dislocation of the hip can only be presumed. There is no “gold standard” for diagnosis during the newborn period [**2**]. 

In 2002, AIUM developed a guideline, to assist practitioners performing sonographic studies for the detection of developmental dysplasia of the hip. AIUM suggested that ultrasound, if available, was the preferred method for the diagnosis imaging of the immature hip. Also, there are no absolute contraindications for the ultrasound of the infant hip for DDH [**3**]. 

The recommendations for screening newborn infants for developmental dysplasia of the hip vary from country to country. Several countries, including Austria, Germany, and Switzerland have introduced the National Screening of Neonatal Hips by Ultrasound [**4**].

In June 2015, the American Academy of Pediatrics recommended ultrasonography in the screening process of developmental dysplasia of the hip in its Technical Report [**5**]. 

Compared to most European countries, in Romania this pathology continues to be discovered in old age. Therefore, the cumulative number of cases of dislocations in children up to 18 is up to 7200 patients (requiring medical care). 

The cost of an operation to treat a case with hip dislocation reached 40-80.000 Euros in the European Union countries. The cost of hip arthroplasty is of approximately 20.000 per patient, totaling about 8 million per year. The total annual costs for the treatment of hip dislocation development in Romania were estimated at 24 million annually.

Also, the early detection of DDH can enable less invasive and potentially more effective corrective procedures [**6**,**7**]. Using indirect comparisons, some studies suggested that an earlier diagnosis was associated with better results. Other studies observed a decrease in the operative rate [**8**,**9**,**10**]. 

## Objectives

• The study of the incidence of the disease locally (Valcea County).

• Identifying the risk and predisposing factors involved in the etiopathogeny and particularity of each case study.

• Creating a registry of performed hip ultrasounds.

• Logging details of infants who have a certain degree of DDS, treatment and follow up in time.

• Cost reduction.

### Materials and methods

A retrospective study enrolling newborns between 04.2013 and 02.2015 was conducted in the Neonatal Department of Valcea County Emergency Hospital. All the patients received standard assessments, correspondent with their medical history recorder, physical examination, and ultrasonography of both hips by using the Graf technique.

### Data collection

The information contained in the observation sheets of the newborns, (all the medical data of the newborns gathered in an electronic database) was collected, including the medical history, clinical examination, and Graf’s ultrasound. The following parameters were analyzed: quantitative variables (birth weight and age at first ultrasound examination) and qualitative variables (area of origin, sex, type of ultrasound, gestational age).

### Methods

**Fig. 1:**
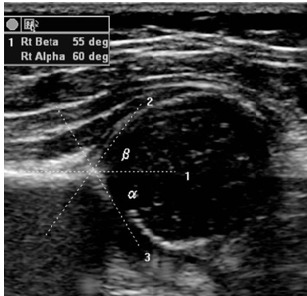
**Fig. 1**Normal infant hip ultrasound

**Fig. 2:**
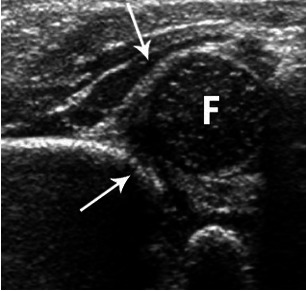
**Fig. 2**Hip showing moderate dysplasia. Arrows point to the acetabulum

Ultrasonography was performed by a neonatologist with Graf Infant Hip Ultrasound Couse diploma with a 7.5 MHz transductor, and by using the Graf method (the patient was placed in the lateral decubitus position). The alpha angle corresponding to the inclination of the bony acetabulum and the beta angle corresponding to the inclination of the cartilaginous acetabulum on a coronal view of the hip joint were calculated and classified (**Fig. 1, 2**). 

In the Graf classification, type I or fully hips were normal. The alpha angle was > 60º. Type II hips represented physiologic immaturity. The alpha angle was 50 to 59º and the beta angle < 77º. Infants older than three months required treatment to prevent further deterioration. Hips with an angle of 49º or less were defined as having a pathological development and are classified as type IIc, D, IIIa, IIIb or IV [**11**,**12**]. 

From the Graf angles measured on these images, the Graf types were assigned to each hip (**Table 1**).

**Table 1:**
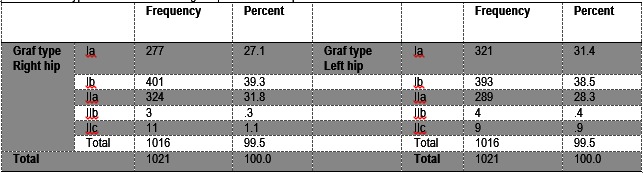
**Table 1**Graf types distribution of the right hip and the left hip

Type Ia, Ib and IIa were considered as having a low risk of developing DDH, type IIb borderline, for which they needed rescanning in a couple of weeks and IIc with DDH. 

## Results

Of the 1021 infants in this study, 509 (49,56%) were females and 515 (50,44%) males. 560 patients (54,85%) were from urban environment and 461 (45,15%) from the country side. 

A total of 27 (20 with IIc and 7 with IIb) newborns had abnormal ultrasound examination. This gave a frequency of 2.64%. 14 (1,4%) had abnormal coverage of the femoral head on the right side, while 13 (1,3%) patients on the left side.

Type of delivery

Regarding the type of delivery, 509 patients (49,8%) had a cesarean section and 512 (50,2%) a normal birth.

Right hip

Of all the cesareans sections, 448 children had a cephalic presentation. 445 had a medium low score of developing DDH (being classified according to Graf in I, IIa and IIb categories) and 3 children were placed in category IIc, being diagnosed with DDH. No child was framed in the category IIb (borderline). 3 out of 53 children with a breech presentation were diagnosed with DDH.

In terms of normal parturition, 494 children had a cephalic presentation and 14 had a breech presentation. In case of cranial presentation, most children (487) had a lower risk of developing DDH, 3 were borderline and 4 children were diagnosed with DDH. Regarding the breech presentation, 13 children had a low-risk and one child was diagnosed with DDH (**Table 2**).

**Table 2:**
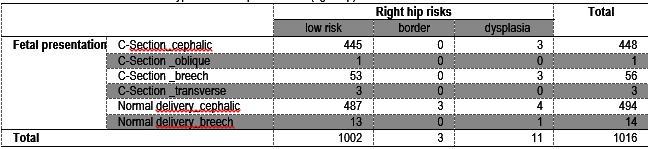
**Table 2**Correlation between Graf types and fetal presentation (right hip)

Left hip

Ultrasound of the left hip diagnosed 3 out of 9 infants with cephalic presentation with DDH; 4 had a breech presentation (C-section) and 2 had a cephalic presentation (normal parturition 4 children were borderline: one with C-section, cephalic presentation and 3 with a normal birth - cephalic presentation (**Table 3**).

**Table 3:**
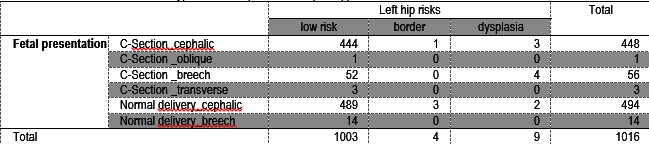
**Table 3**Correlation between Graf types and fetal presentation (left hip)

We have applied Pearson Chi-Square Tests for non-parametric variables. After the analysis of the data, a cephalic delivery was found to be associated with the lower risk for DDH (with p=0,045 for the right hip and p=0.001 for the left hip) (**Table 4**).

**Table 4:**
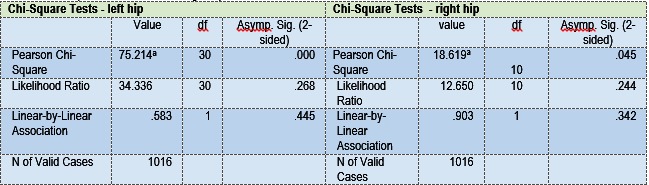
**Table 4**Chi-Square tests for left and right hips

Parturition

542 (53.1%) mothers were at their first birth. It was observed that there was correlation between the first birth and the DDH on the right hip, but without any statistical significance.

Gestational age and Birth weight

The descriptive analysis showed that the minimum gestational age was 22 weeks and the maximum was 50 weeks, with an average of 38,39 weeks. The minimum birth weight was 600 grams, maximum 4800 grams, with an average of 3138,31 grams. 

Anova test was used to establish the correlation between the gestational age/ birth weight and the Graf type of DDH.

**Fig. 3:**
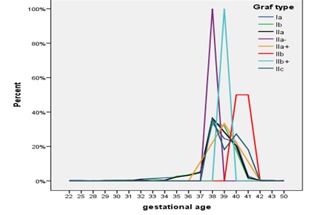
**Fig. 3**Correlation between gestational age and Graf type

- In case of a natural birth, the risk of developing DDH was higher in children with an increased birth weight, with a p=0,022 (**Table 5**).

- The increased gestational age was an incriminating factor in the occurrence of DDH (p=0,05) 

- - In patients with low risk, the 38 weeks gestational age was associated with the lowest risk of developing DDH (**Fig. 3**). 

**Table 5:**
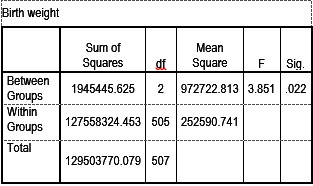
**Table 5**ANOVA for birth weight

### Discussion

DDH is an important term because it accurately reflects the biological features of the disorders and the susceptibility of the hip to become dislocated at various times. Dislocated hips will always be diagnosed later in infancy and childhood because a dislocated hip is detectable at birth and hips continue to dislocate throughout the first year of life [**13**]. According to the International Hip Dysplasia Institute, hip dysplasia is approximately 12 times more frequent if there is a family history of it. Also, the intrauterine baby position can increase pressure on the hips. It is thought that babies in a normal position have more stress on the left hip than on the right hip. Babies in a breech presentation are more likely to have a hip instability than babies in a normal womb position [**14**,**15**,**16**].

A study made in Austria showed that the breach presentation, female sex, primiparity, high birth weight, older maternal age, postmaturity are confirmed as risk factors for DDH [**17**]. In primiparity, the pressure of a previously un-distended uterine wall would restrict the fetus. It is not clear how increased maternal age resulted in suboptimal fetal accommodation, but a preterm birth (< 37 weeks of gestation) and a multiple birth both reduce the risk of DDH [**17**,**18**].

Another factor to consider is whether the external cephalic version of the breech presentation at 38 weeks of gestation would result in a reduction in risk of DDH [**17**].

In the study made in 1997, Annabelle Chan and co. said that the breech presentation, oligohydramnios, female sex and primiparity were confirmed as risk factors for DDH and they had to be used as indicators for repeated screening at 6 weeks of age and whenever possible in infancy [**19**,**20**].

In Romania, the incidence of DDH is still high and this pathology is still discovered in older age. In this retrospective study, 27 of 1021 patients had an abnormal coverage of the femoral head at ultrasound examination and 20 of 27 patients were classified by Graf type in category II C, when they were diagnosed with DDH.

Concerning the type of delivery, it seems that the cephalic presentation is associated with the lower risk for DDH. The main risk factors confirmed for DDH are increased birth weight, increased gestational age, and primiparity. 

The authors used hip ultrasonography to calculate the femoral head coverage in this study and that seems to be the most efficient method for an early diagnosis of DDH. The ultrasound examination is a useful tool to confirm the DDH presence.

## Conclusions

Graf’s method can provide a qualitative and quantitative evaluation for the hips of infants. It is an effective mean of investigation (investigational tool) in developmental dysplasia and it is also useful for the early treatment decision and follow-up of DDH.

The main advantages of ultrasonography include its ease of use, its freedom from ionizing radiation, its capacity to reveal non-body structures and its capacity of evaluating the progress of therapy.

It would be reasonable to target an educational programme to all medical practitioners, which must include information about DDH, the importance of early detection, the risk factors, the methods of screening and the need for repeated screening [**17**,**21**].

### Sources of Funding

This work received financial support through the project entitled “CERO – Career profile: Romanian Researcher”, grant number POSDRU/159/1.5/S/135760, cofinanced by the European Social Fund for Sectoral Operational Programme Human Resources Development 2007-2013.

**Disclosures**

None
